# Cytomegalovirus induces HLA-class-II-restricted alloreactivity in an acute myeloid leukemia cell line

**DOI:** 10.1371/journal.pone.0191482

**Published:** 2018-01-29

**Authors:** Michael Koldehoff, Monika Lindemann, Stefan R. Ross, Ahmet H. Elmaagacli

**Affiliations:** 1 Department of Bone Marrow Transplantation, West German Cancer Center, University Hospital of Essen, Essen, Germany; 2 Institute for Transfusion Medicine, University Hospital of Essen, Essen, Germany; 3 Institute of Virology, University Hospital of Essen, Essen, Germany; 4 Department of Hematology and Stem Cell Transplantation, Asklepios Clinic St. Georg, Hamburg, Germany; University of St Andrews, UNITED KINGDOM

## Abstract

Cytomegalovirus (HCMV) reactivation is found frequently after allogeneic hematopoietic stem cell transplantation (alloSCT) and is associated with an increased treatment-related mortality. Recent reports suggest a link between HCMV and a reduced risk of cancer progression in patients with acute leukemia or lymphoma after alloSCT. Here we show that HCMV can inhibit the proliferation of the acute myeloid leukemia cell line Kasumi-1 and the promyeloid leukemia cell line NB4. HCMV induced a significant up-regulation of HLA-class-II-molecules, especially HLA-DR expression and an increase of apoptosis, granzyme B, perforin and IFN-γ secretion in Kasumi-1 cells cocultured with peripheral blood mononuclear cells (PBMCs). Indolamin-2,3-dioxygenase on the other hand led only to a significant dose-dependent effect on IFN-γ secretion without effects on proliferation. The addition of CpG-rich oligonucleotides and ganciclovir reversed those antiproliferative effects. We conclude that HCMV can enhance alloreactivity of PBMCs against Kasumi-1 and NB4 cells in vitro. To determine if this phenomenon may be clinically relevant further investigations will be required.

## Introduction

Human cytomegalovirus (HCMV) is a member of the betaherpesvirus family with a moderate seroprevalence among adults [1]. In immunocompromised hosts like newborn’s, recipients of stem cell transplants or other immunodeficient individuals CMV reactivation commonly manifests as a life-threating disease affecting different organ systems, whereas symptomatic infections of healthy individuals are rare. HCMV survival is enhanced by immunosuppression and by reduction of intragraft MHC-linked antiviral T cell responses in allogeneic hematopoietic stem cell transplantation (alloSCT) [2]. Variability in HCMV genomic sequences affects cellular tropism and replication [3]. Moreover, in transplant recipients asymptomatic HCMV viremia often precedes invasive HCMV infections [4]. HCMV reactivation was previously thought to be associated with a worse transplant outcome [5], but recently it was demonstrated that HCMV reactivation correlates with inhibition of malignant progression in patients with acute myeloid leukemia (AML) and other haematological diseases after alloSCT [6–8]. Dynamic T cell and NK cell responses are documented in the context of early and late HCMV infection, in particular following alloSCT and solid organ transplantation [9–11]. Although immune reconstitution after alloSCT has been thoroughly examined, HCMV diversity and its possible effects on molecular pathways influencing clinical outcomes is poorly understood [12–14]. This study assesses effects of HCMV and acute leukemic cells on nonspecific and specific responses that augment T cell or other alloimmune activities by using *in vitro* assays such as flow cytometry and ELISpot.

## Methods and methods

### Cells

All cell lines except Kasumi-1 were purchased and maintained as instructed by the DSMZ, Braunschweig, Germany. The AML cell line Kasumi-1 was a generous gift from Dr. Nanao Kamada (Hiroshima, Japan). This cell line was established from the peripheral blood of a 7 year-old boy suffering from AML. Dr. Kamada has given the Kasumi-1 cell line to one author (Dr. Elmaagacli) as a gift for scientific trials to the University of Essen, so we received the oral consent and informed consent for the derivation and use of the Kasumi-1 cell line. Furthermore, the cell line Kasumi-1 is also available by the DSMZ. Peripheral blood mononuclear cell (PBMC) samples were collected from healthy volunteers after informed consent in accordance with institutional guidelines.

### HCMV infection

For infection we used the HCMV strain AD169 (ATCC-VR-538 American Type Culture Collection, Manassas, VA, USA), as described [15]. Cell-free virus stock and infections were prepared as previously described [16]. All infections were conducted at a multiplicity of infection (MOI).

### In vitro assays

Kasumi-1 cells without and with prior HCMV infection were tested for their viability and in vital cells proliferation and the secretion of IFN-α were assessed. To determine proliferation 12,500–400,000 Kasumi-1 cells were grown in quadruplicates for six days in 200 μl cell culture medium (RPMI 1640, GIBCO, Life Technologies, Paisley, UK, with 10% of inactivated pooled human serum) per well of microtiter plates (37°C, 5% CO_2_). To determine IFN-α secretion by the ELISpot 25,000–800,000 Kasumi-1 cells were grown in duplicates for two days using the same medium.

To assess proliferation, the cultures were labeled for the final 16 hours with 37 MBq H3 thymidine per well (TRA.120, specific activity 5 Ci/mmol, GE Healthcare, Buckinghamshire, UK) [17]. The cultures were then harvested (Harvester 96, Tomtec, Hamden, CT, USA) onto filter pads (Wallac, Turku, Finland), and the incorporated radioactivity was quantified by liquid scintillation counting (1450 Microbeta Trilux, Wallac). Results were given as counts per minute (CPM) values of H3 thymidine uptake. To assess apoptosis and viability cells were labelled with monoclonal antibodies (Annexin V, 7-AAD and CD45, from Coulter, Krefeld, Germany) according to the manufacturer´s protocol and analyzed by flow cytometric assays (FACSCalibur, Becton Dickinson [BD], Heidelberg, Germany) as previously described [15]. Granzyme B, perforin and IFN-γ secreting cells were determined by ELISpot as previously described [18]. The IFN-α ELISpot assays followed the same procedure but used clone MT1/2/5 (15 μg/ml) as coating and clone MT2/4/6 (1 μg/ml) as detection antibody (both from Mabtech, Nacka, Sweden).

### Co-culture experiments

Kasumi-1 cells without and with HCMV infection were co-cultured with PBMC in 200 μl of cell culture medium. To determine proliferation or cytokine secretion 50,000 or 150,000 cells each were cultured for six days or three days, respectively. In each set of experiments, PBMC of 2 to 3 healthy controls were co-cultured with Kasumi-1 cells. The PBMC of each healthy control were tested in a separate assay. Kasumi-1 cells were also grown alone and their spontaneous proliferation and cytokine secretion was determined. Flow cytometry was performed on Kasumi-1 cells and PBMC that were grown in 2 ml of cell culture medium separated by cell culture inserts (0.4 μm pore size, ThinCerts, Greiner Bio-One, Frickenhausen, Germany) which allowed easy separation of both cell types. Human leukocyte antigen (HLA) class I and class II expression on the surface of Kasumi-1 cells was determined after two days of cell culture by flow cytometry (FACSCalibur, BD), using either PE-labelled antibodies against HLA-A, B and C (w6/32, Serotec, Puchheim, Germany) or FITC-labelled antibodies against HLA-DP, DQ and DR (WR18, Serotec, or HLA-DP, HLA-DQ and HLA-DR, BD). Unlabelled Kasumi-1 cells were always measured in parallel to determine their autofluorescence. This value was considered background which was subtracted. The analysis was performed applying a linear format to measure channel fluorescence intensities as numerals and to calculate median fluorescence intensity (MFI) values.

### Mixed lymphocyte cultures

One-way mixed lymphocyte cultures (MLC) of PBMC were performed using 50,000 responder cells (RC) and 50,000 stimulator cells (SC) (irradiated with 30 Gy) to assess lymphocyte proliferation or using 150,000 cells each to assess numbers of IFN-γ secreting cells. Triplicate cultures were grown for six days and duplicate cultures for three days, respectively, and proliferation and cytokine secretion were determined as detailed above. Parallel experiments were performed without and with 1 μM and 10 μM indolamin-2,3-dioxygenase (IDO, Enzo Life Sciences, Lörrach, Germany).

### Effect of CpG and ganciclovir on co-cultures

Kasumi-1 or acute promyeloid NB4 cells without and with HCMV infection were co-cultured with PBMC, using 7,500 and 75,000 cells, respectively. Quadruplicate cultures were grown for five days. H3 thymidine was added on day 5 and cell proliferation was determined on day 6. Parallel tests were run with and without the addition of 3 μg/ml CpG-rich Oligodeoxynucleotides (CpG-rich ODNs, see [14]) and/or 5 μg/ml ganciclovir (Cymevene^®^, Hoffmann-La Roche, Basel, Switzerland).

### Statistical analysis

*In vitro* experiments were analyzed by student’s t-test. Cell number dependent proliferation or IFN-α secretion of Kasumi-1 cells without and with prior HCMV infection was compared by one- and two-way ANOVA (GraphPad Prism version 5.03 for Windows (GraphPad Prism Software, La Jolla, CA), and EZR software for medical statistics from Y. Kanda [19].

## Results and discussion

**Fig 1:**
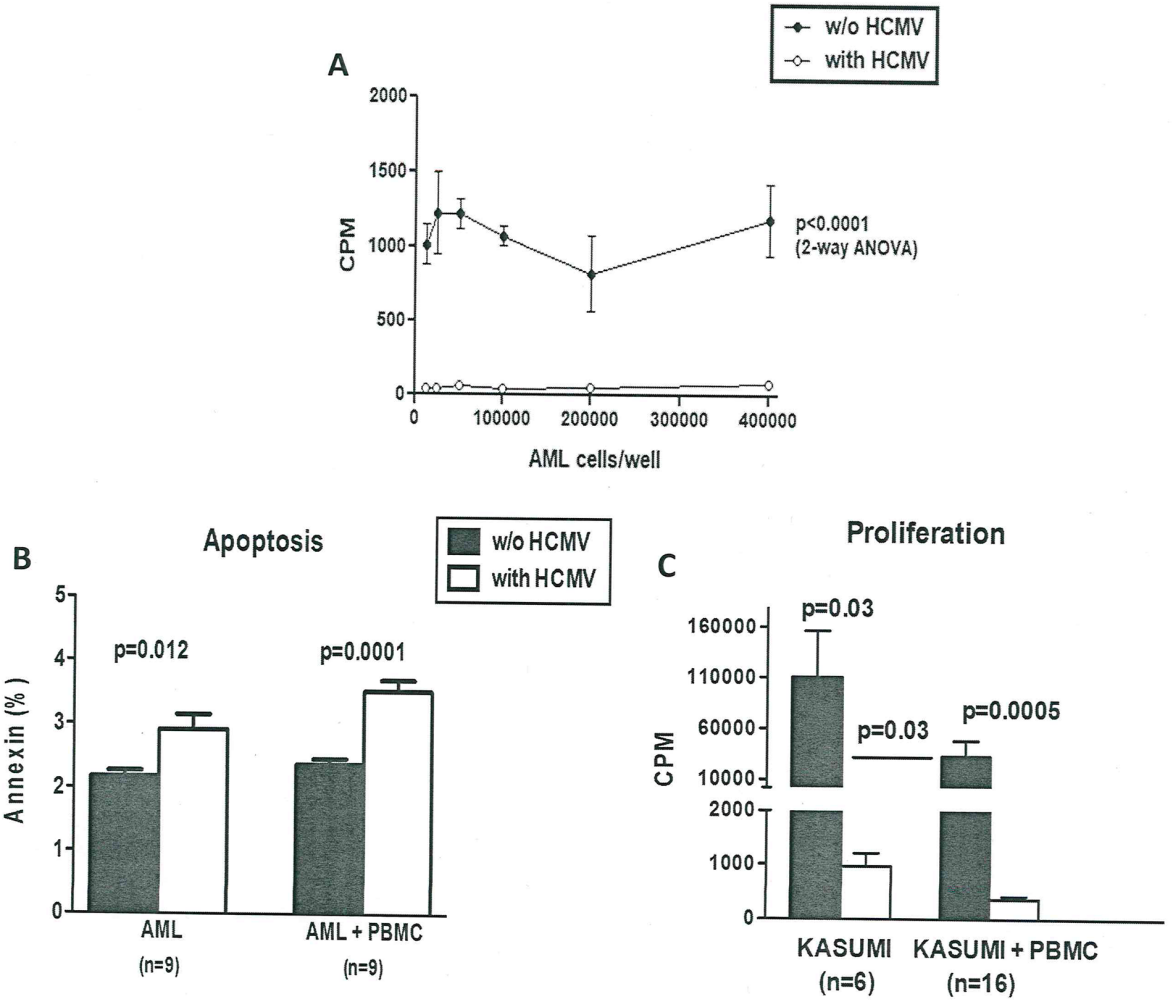
A: HCMV infection inhibits proliferation of AML cells. 12,500–400,000 AML cells were cultured for six days in the absence or presence of HCMV infection. Proliferation was measured by H3 thymidine incorporation. Mean and SD of counts per minute (CPM) are presented. B: Apoptosis of HCMV-infected AML cells. The rate of apoptosis in AML cells 48 h without vs. with HCMV-infection and apoptosis in AML cells co-cultured with PBMC measured by annexin detection (%). C: Proliferation of HCMV-infected Kasumi-1 cells and co-culture experiments of Kasumi-1 and PBMC. Proliferation of Kasumi-1 cells without vs. with HCMV infection and co-cultured with PBMC was analyzed by H3 thymidine uptake and results were given as counts per minute (CPM).

Alloreactivity in alloSCT involves either major and minor histocompatibility complex (MHC) antigens as well as other poorly defined antigens. HCMV reactivation is frequently observed after alloSCT, and usually resolves soon by early preemptive treatment with nucleoside antiviral agents such as ganciclovir or foscavir [20]. We analyzed proliferation in HCMV-infected AML cells and found that HCMV infection of AML cells almost completely stopped proliferation of a wild range of input cell numbers (12,500–400,000) compared to uninfected AML cells with a mean value of 1,092 CPM (SD 156 and range 826–1,225 CPM) (Fig 1A). Furthermore, we found a significant induction of apoptosis by input cell number (200,000–400,000) after 72 h in HCMV-infected acute myeloid cells of 2.9% ± 0.7 (range 2.0–4.1%, p<0.012) compared to uninfected AML cells with a spontaneously apoptosis rate of 2.2% ± 0.3 (Fig 1B). Co-culture experiments showed that PBMC had different effects on Kasumi-1 cells without vs. with HCMV infection. Cell proliferation was significantly lower if Kasumi-1 cells were infected with HCMV (3.4-fold, p<0.03) and the proliferation of Kasumi-1 cells without vs. with HCMV infection was also significantly lower when co-cultured with PBMCs (382 vs. 32,860 CPM, i.e. 86-fold, p<0.0005). PBMCs strongly inhibited proliferation of Kasumi-1 cells which was more effective if Kasumi-1 cells were infected with HCMV (Fig 1C). Infection with HCMV also induced apoptosis in the Kasumi-1 cells co-cultured with PBMCs of 3.5% ± 0.5 (range 2.9–4.3%, p<0.0001) compared to uninfected AML cells co-cultured with PBMC with a spontaneously apoptosis rate of 2.4% ± 0.3 (Fig 1B). No significant differences in early apoptosis were seen in PBSCs with or without HCMV infection.

**Fig 2:**
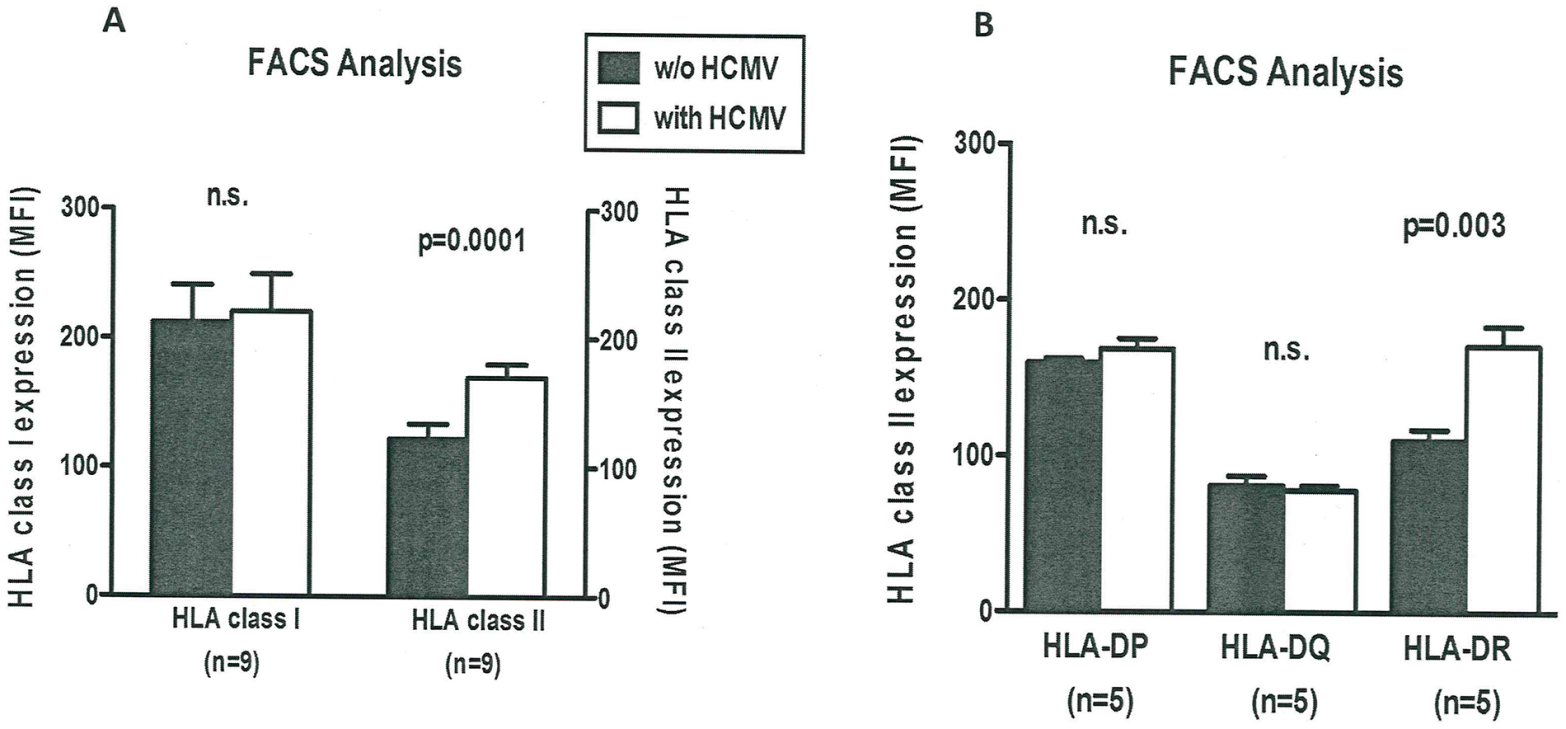
A: Flow cytometry analysis of Kasumi-1 cells and PBMC. Expression of HLA class I and class II on Kasumi-1 cells without vs. with HCMV infection, both co-cultured with PBMC, was analyzed by flow cytometry. MFI, median fluorescence intensity, (w/o), without. B: HLA class II analysis of Kasumi-1 cells and PBMC. Expression of HLA-DP, HLA-DQ and HLA-DR on Kasumi-1 cells without vs. with HCMV infection, both co-cultured with PBMC, was analyzed by flow cytometry.

We next assessed by FACS analysis whether HCMV infection affected HLA class I or class II expression in co-culture experiments with Kasumi-1 cells and PBMCs. Kasumi-1 cells with vs. without HCMV infection displayed significantly higher HLA class II expression after co-culture with PBMC (Fig 2A). However, early HLA class I expression was not significantly influenced by HCMV infection indicating that early interactions between Kasumi-1 cells (with vs. without HCMV infection) and PBMC may be mediated via HLA class II. In order to confirm the early mechanism of HLA class II expression in HCMV-infected cells we measured differences in HLA-DP, HLA-DQ and HLA-DR expression. We found either no significant difference in early HLA-DP expression and HLA-DQ expression with vs. without HCMV infection after co-culture with PBMC (HLA-DP: 168 ± 15 vs. 160 ± 6 MFI and HLA-DQ: 78 ± 6 vs. 81 ± 13 MFI), whereas early HLA-DR expression significant increased with vs. without HCMV infection after co-culture with PBMC (171 ± 28 vs. 110 ± 16 MFI, p<0.003) (Fig 2B). When we increased the number of samples the effect of HLA-DR expression could be confirmed again (HLA-DR: 143 ± 56 vs. 93 ± 36 MFI, p<0.03). HCMV-infected myeloid cells carry the pathogen or pathogen components to arbitrate several immune mechanisms and the type of immune response is highly dependent on the local inflammatory environment [21]. This study shows that HCMV inhibits the proliferation and induces apoptosis in AML cells. Moreover, co-culture with PBMC during HCMV infection increased apoptosis of AML cells. Induction of apoptosis in HCMV-infected cells is unexpected and unusual because HCMV possesses different strategies to prevent apoptosis in infected cells in order to maintain viral replication [22]. Gabaev and coworkers found that the HCMV protein UL.11 altered the cellular protein tyrosine phosphatase CD45 resulting in functional paralysis of immune cells, especially restricting T-cell proliferation and signal transduction [23]. Despite the heterogeneity of CD45 expression in AML cells, CD45 expression is usually lower than on normal lymphoid, monocytic and myeloid cells [24]. Therefore, the lower CD45 expression in HCMV-infected AML cells may alter the distribution of HCMV encoded proteins and suppress the numerous strategies of apoptosis inhibition by HCMV.

Different combinations of pattern recognition receptors (PRR) play a role in the defenses mechanisms to HCMV infection such as Toll-like receptors (TLR), especially TLR 2 or endosomal TLR7/9 and cytoplasmic sensors. Those PRR regulate important functions in host cells such as activation or modulation of type I IFN genes or other signaling molecules in the context of HCMV infection [25]. Variability in HCMV genomic sequences contributes to differences in cellular tropism and replication whereas the cellular tropism of HCMV also reflects various nonspecific and specific receptors involved in HCMV infection. HCMV encodes different proteins that appear to interfere with host immune responses including alteration of MHC class I and II expression on antigen-presenting cells (APCs) and other immune modulatory effects [26]. HCMV infection has been shown to alter immune responses after transplantation by modifying the phenotype of T and NK cells, their receptor repertoire and function [12,27–28]. Immune recovery of T cell subsets post alloSCT correlates with control of HCMV reactivation and decreased relapse rates in leukemia patients [15,29].

**Fig 3:**
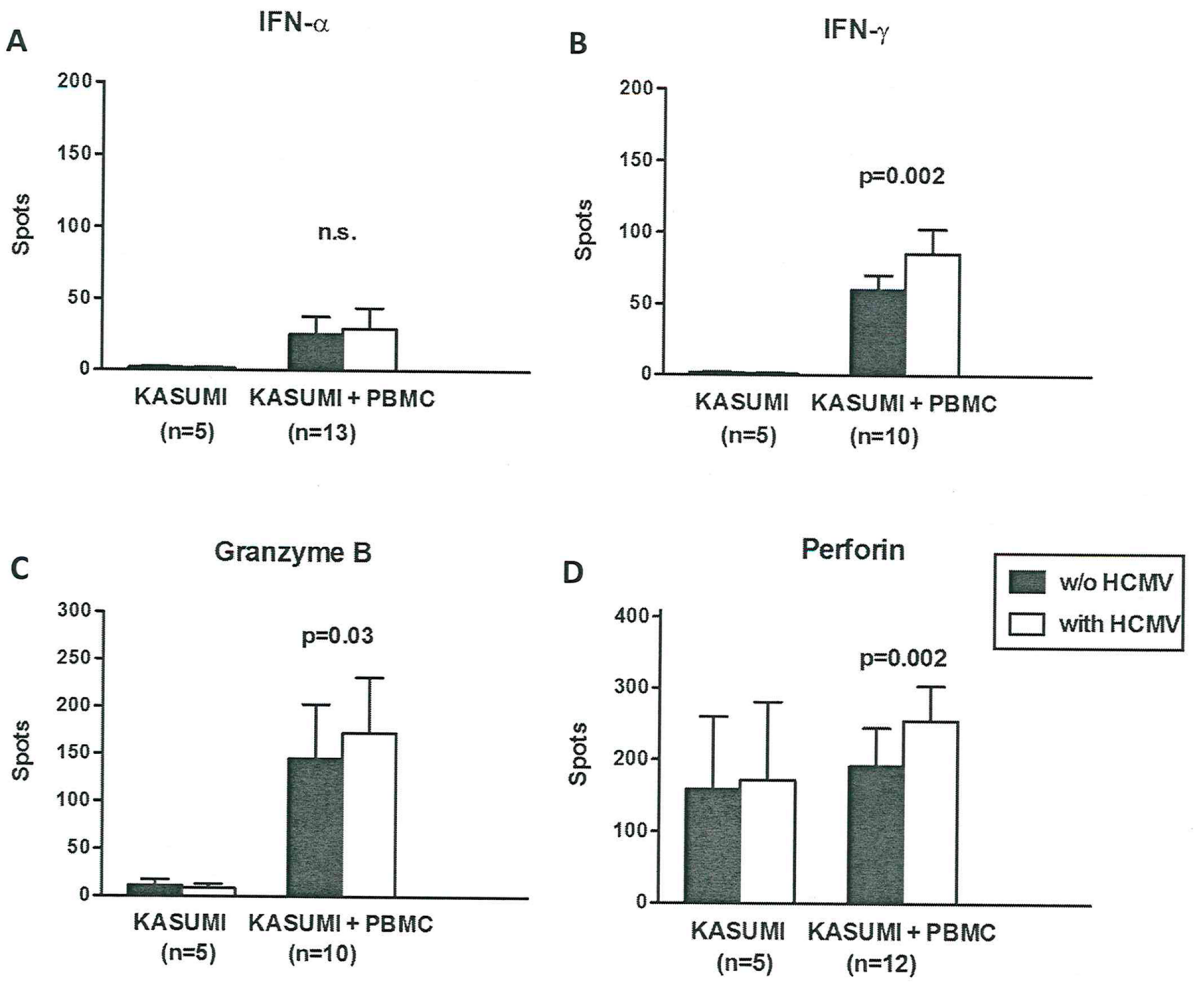
A: Secretion of IFN-α in co-culture experiments of Kasumi-1 cells. Panel (A) to (D) display co-culture experiments of Kasumi-1 cells without vs. with HCMV infection and PBMC of healthy controls. IFN-α (panel A) was determined by ELISpot assay. ELISpot results are given as spots per well. Kasumi-1 cells without and with HCMV infection were compared by Wilcoxon matched pairs test, Kasumi-1 cells and Kasumi-1 cells plus PBMC were compared by Mann-Whitney test. B: Secretion of IFN-γ in co-culture experiments of Kasumi-1 cells. IFN-γ (panel B) was determined by ELISpot assay. C: Secretion of granzyme B in co-culture experiments of Kasumi-1 cells. Granzyme B (panel C) was determined by ELISpot assay. D: Secretion of perforin in co-culture experiments of Kasumi-1 cells. Perforin (panel D) was determined by ELISpot assay, (w/o), without.

In order to confirm the mechanism of HLA class-II-restricted alloreactivity we performed additional co-culture experiments to evaluate the cytokine profile of PBMC reacting against HCMV-infected Kasumi-1 cells. Spontaneous IFN-α secretion by Kasumi-1 cells (AML FAB 2) without HCMV infection resulted in 2.0 ± 1.6 spots as determined by ELISpot assay. In contrast, in Kasumi-1 cells infected with HCMV we found 1.6 ± 1.8 spots. IFN-α secretion was therefore nearly absent in Kasumi-1 cells cultured alone and only low levels were produced during co-culture with PBMCs, independent from HCMV infection (Kasumi-1 with PBMC with HCMV vs. w/o: 38 ± 54 vs. 26 ± 45 spots) (Fig 3A). Secretion of IFN-γ, granzyme B and perforin was not altered when Kasumi-1 cells were HCMV-infected (IFN-γ: 1.8 ± 0.8 vs. 1.2 ± 0.4 spots, granzyme B: 10.8 ± 14.0 vs. 8.2 ± 9.9 spots, perforin: 159 ± 227 vs. 172 ± 243 spots, all not significant). By co-culture with PBMCs the majority of IFN-γ and granzyme B secretion could be attributed to PBMCs while perforin was released by both PBMCs and Kasumi-1 cells (Fig 3B, 3C and 3D). Secretion of IFN-γ and granzyme B was increased when Kasumi-1 cells were HCMV-infected (IFN-γ: 86 ± 54 vs. 61 ± 33 spots, p<0.002, granzyme B: 172 ± 186 vs. 145 ± 182 spots, p<0.03). Perforin levels were also significantly elevated indicating that HCMV infection induced allogeneic PBMCs to mount an increased cytotoxic response against Kasumi-1 cells (perforin: 255 ± 168 vs. 192 ± 185 spots, p<0.002, (Fig 3D)). Pachnio et al. investigated the phenotypic and transcriptional profile of HCMV-specific CD4+ T cells in healthy donors with HLA class II-peptide tetramers [30]. The authors report that HCMV-specific CD4+ T cells display a highly differential effector memory phenotype and express a range of cytokines including IFN-γ, TNF-α, cytotoxic enzymes such as granzymes B, H, A, granulysin and perforin, the chemokines like CCL3 (MIP-1α), CCL4 (MIP-1β), CX3CR1 and a marked overexpression of the ADRB2, the gene encoding the β2-adrenergic receptor [23]. The latter observation is suggestive of an important link between the sympathetic nervous system and the immune system. It is likely that NK cells and NKG2 epitopes are potential alternative costimulatory molecules and can synergized with TCR-dependent activation of CMV-specific T cells to enhance a range of effector functions [31–32].

**Fig 4:**
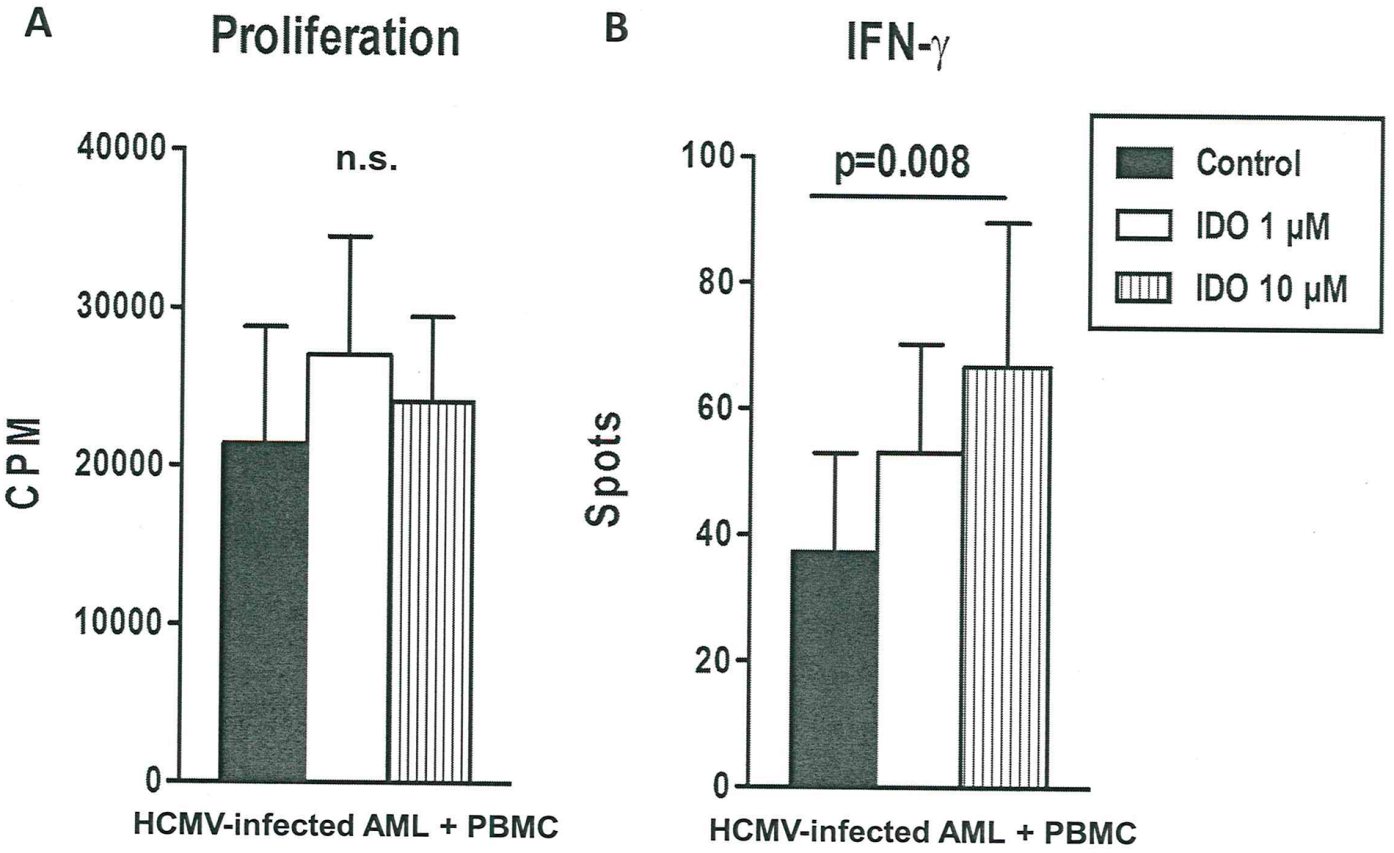
A: Alloreactivity-mediated proliferation of indolamin-2,3-dioxygenase-treated HCMV-infected AML cells co-cultured with mixed lymphocyte cultures. HCMV-infected AML cells co-cultured with mixed lymphocyte cultures from healthy controls were performed without and with 1 μM and 10 μM indolamin-2,3-dioxygenase (IDO). Panel (A) shows proliferative responses determined by H3 thymidine uptake (n = 3 experiments) and proliferative responses are presented as counts per minute (CPM). B: IFN-γ secretion by indolamin-2,3-dioxygenase-treated HCMV-infected AML cells co-cultured with mixed lymphocyte cultures. Panel (B) displays IFN-γ secretion determined by ELISpot, ELISpot results are given as spots per well. One-way-ANOVA (Friedman test) indicates that the groups were significantly different (p = 0.008) in terms of IFN-γ secretion.

We next evaluated the effect of indolamin-2,3-dioxygenase (IDO) on HCMV-infected AML cells in mixed lymphocyte cultures and observed that the addition of IDO did not alter alloreactivity-mediated proliferation in Kasumi-1 cells. With regard to the effects on IFN-γ the addition of IDO led to a significant (p<0.008) dose-dependent induction of IFN-γ secretion indicating that alloreactivity was not repressed by IDO in HCMV-infected Kasumi-1 cells (Fig 4B).

IDO is an intracellular enzyme that catalyzes the degradation of the amino acid tryptophan to kynurenine, and is able to suppress T cell proliferation and is considered to be involved in tolerogenic function. Munir et al. detected a trend between class-I and class II-restricted IDO responses and an association between IDO-specific CD4+ T cells and CD8+ CMV-responses. They showed that IDO-specific CD4+ T cells released IFN-γ as well as TNF-α. IDO-specific T cells may participate in immune-regulatory networks where the activation of pro-inflammatory IDO-specific CD4+ T cell responses could reduce the immunosuppressive effects of the IDO-protein, which otherwise leads to the effect of early IDO expression in maturing antigen-presenting cells [33]. HCMV has been shown to induce IDO expression in monocytes, which might present an advantage to CMV-infected monocytes attempting to escape T cell responses [34]. HCMV is also known to alter the T cell repertoire of HCMV-specific circulating memory T cells [35].

**Fig 5:**
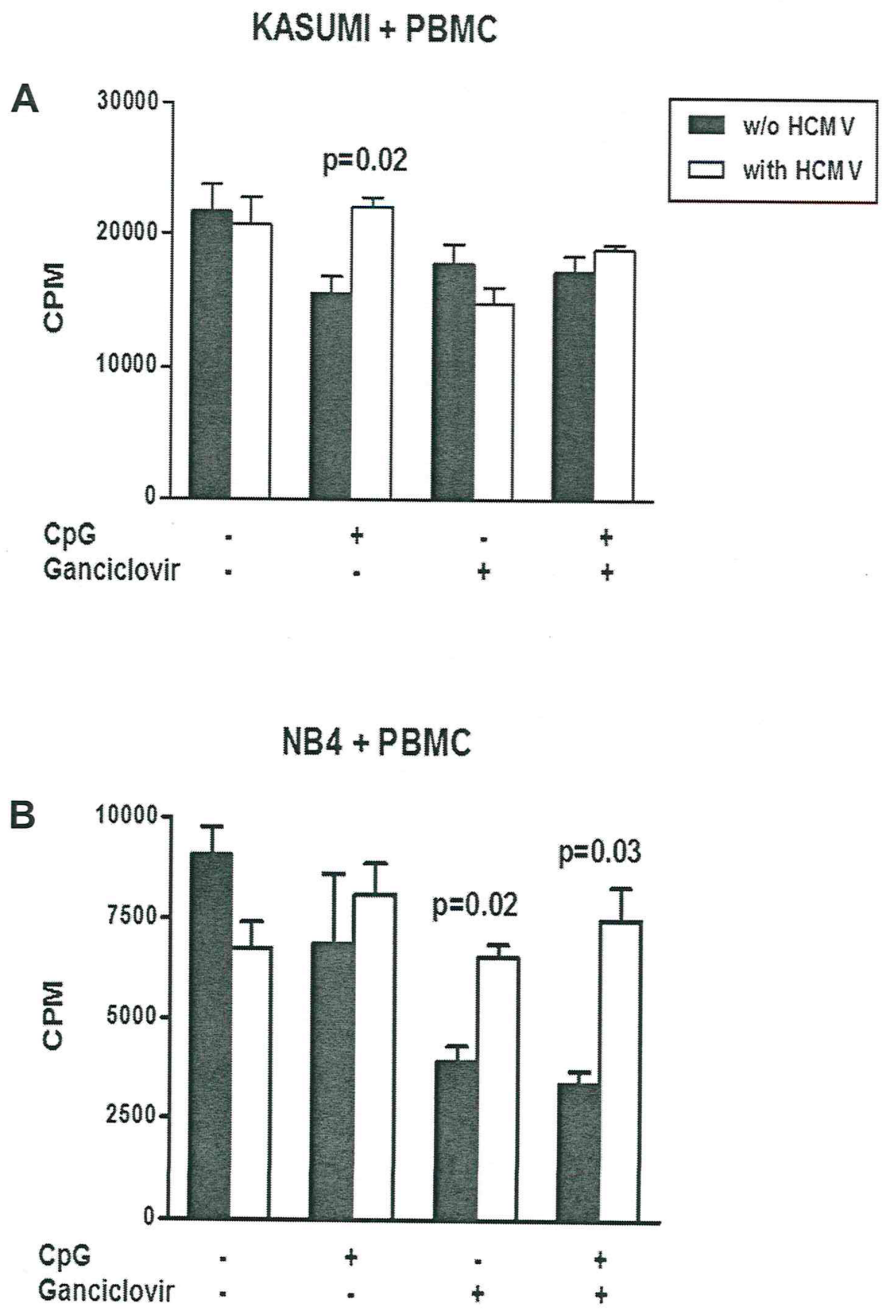
Effect of CpG and ganciclovir on proliferation of HCMV-infected leukemia cells. Kasumi-1 or NB4 cells without (w/o) and with HCMV infection were co-cultured with PBMC without and with CpG-rich ODNs and/or ganciclovir and cell proliferation was measured by H3 thymidine uptake. The addition of CpG-rich ODNs to the co-cultures resulted in significantly higher proliferation of Kasumi-1 cells with vs. without CMV infection (panel A). Ganciclovir and the combination of CpG-rich ODNs and ganciclovir led to significantly increased proliferation in HCMV infected NB4 cells (panel B). Proliferative responses are given as counts per minute (CPM). Kasumi-1 or NB4 cells without and with HCMV infection were compared by Wilcoxon matched pairs test.

We found that the addition of CpG-rich ODNs to co-cultures of Kasumi-1 cells and PBMCs resulted in significantly higher proliferation of Kasumi-1 cells with vs. without HCMV infection, i.e., antiproliferative effects of HCMV infection were reverted by CpG-rich ODNs (Fig 5A). A similar phenomenon was observed for NB4 cells but did not reach statistical significance. Given the low expression of HLA-DR epitopes by Kasumi-1 cells and the absence of HLA-DR epitopes on NB4 cells it is possible that those difference can be attributed to the differential expression of myeloid markers and autoactivation of HLA class II epitopes. In addition, ganciclovir and the combination of CpG-rich ODNs and ganciclovir led to significantly (p < 0.05) increased proliferation in HCMV infected cells (Fig 4B). Surprisingly, CpG-rich ODNs and ganciclovir increased the proliferation of acute promyeloid NB4 cells, whereas in the acute myeloid Kasumi-1 cells CpG-rich ODNs and ganciclovir application do not stimulate proliferation in the setting of HCMV infection. Melenhorst et al. showed that T cell alloreactivity against non-self peptide–HLA complexes in CD4+ and CD8+ T cells is caused by both naïve and memory T cell compartments. Moreover, virus-specific T cell lines cross-react with allogeneic peptide-MHC complexes [36] and Falkenburg´s group described that the reactivation of CMV-specific responses can be elicited from naïve donor populations [37]. They demonstrated that the probability of sustained generation of primary immune responses by a localized balance between the antigen-specific precursor T cells and regulatory T cells determines the possible strength of the immune response [30]. Although the addition of a stimulating cytokine milieu can be beneficial for the expansion of antigen-specific T cells, off-target proliferation of bystander T cells and NK cells has been observed. Furthermore, T cells recognizing polymorphic peptides derived from proteins encoded by genes selectively expressed in hematopoietic lineages may result in selective graft versus leukemia activity depending on the magnitude and diversity of the alloreactive T cell response [29–30,38].

In conclusion, although several mechanisms of action of immune responses against defined HCMV have been proposed, we found possible allogeneic HLA class-II restricted antiproliferative effects associated with distinct cytokine profiles *in vitro*. However, our findings may be limited to the tested HCMV strain AD169 and it will need to be confirmed if they apply to additional HCMV strains and other herpesvirus. The assessment of cellular immunity against HCMV infected leukemic cells after alloSCT should include evaluation of HCMV-modulated γδ T cells, CD4+ T cells, CD8+ T cells co-expressing NKG2 epitopes, NK cells expressing different KIRs and CMV-specific peptidomes in order to prevent or treat HCMV infection or leukemic relapse in patients after alloSCT.

## Supporting information

S1 File Raw data. Detailed results of all experiments performed.
(XLSX)

S2 File Raw data Kasumi. Detailed results of Kasumi experiments performed.
(PZF)

S3 File Raw data apoptosis. Detailed results of apoptosis experiments performed.
(PZF)

S4 File Raw data alloreaction. Detailed results of alloreaction experiments performed.
(PZF)
